# Exploring the mental healthcare needs of Swiss pre-trial detainees: A pilot investigation of an on-site psychiatric day clinic

**DOI:** 10.3389/fpsyt.2022.924861

**Published:** 2022-07-19

**Authors:** Juliane Gerth, Jérôme Endrass, Michael Weber, Marc Graf, Jay P. Singh, Astrid Rossegger

**Affiliations:** ^1^Department of Psychology, University of Konstanz, Konstanz, Germany; ^2^Department of Research and Development, Office of Corrections and Rehabilitation, Zurich, Switzerland; ^3^Department of Forensic Psychiatry, University of Basel, Basel, Switzerland; ^4^Department of Forensic Psychiatry, University Psychiatric Hospitals, Basel, Switzerland; ^5^Publication Academy Inc., Reston, VA, United States

**Keywords:** mental health, prison psychiatry, pre-trial detention, psychiatric day clinic, prevention of mental crises

## Abstract

**Introduction:**

Research has established that justice-involved individuals experience significant mental health problems. However, mental healthcare in correctional settings is often not sufficiently accessible to meet the demand. Hence, to improve the availability of mental healthcare services, especially for pre-trial detainees, the first Swiss on-site psychiatric day clinic (PDC) was established in 2019. The present cross-sectional observational study aimed to evaluate the need of psychiatric care in pre-trial detention and the PDC's potential to improve it.

**Methods:**

File record data were collected from the Office of Corrections and Rehabilitation of the Canton of Zurich. Differences in primary psychiatric care consultations and psychiatric hospital admissions between pre-trial detainees and sentenced prisoners were examined. In addition, a total cohort of pre-trial detainees of the first 18 months of PDC operations was examined to identify differences between three treatment groups: (1) pre-trial detainees exclusively treated in the PDC (*n* = 41), (2) pre-trial detainees exclusively treated in a psychiatric hospital (*n* = 58), and (3) pre-trial detainees treated in both the PDC as well as a psychiatric hospital (*n* = 16).

**Results:**

In the 5 years before the PDC opened, pre-trial detainees had significantly more primary psychiatric care consultations and were admitted to psychiatric hospitals on significantly more occasions than were sentenced prisoners. In the first 18 months of the PDC, psychiatric hospital admission rates for pre-trial detainees decreased by 18.5% and pretrial detainees exclusively treated in the PDC differed significantly from other treatment groups concerning mental disorder, gender, and alleged index offense. They were more likely to be diagnosed with adjustment disorders and were less likely to be diagnosed with schizophrenia spectrum disorder.

**Conclusion:**

The use of mental health care among pre-trial detainees is significantly more frequent than among sentenced prisoners concerning both primary care and inpatient treatment. Since establishment of the novel on-site PDC admissions to psychiatric hospitals were found to decrease. Data indicates that especially male pre-trial detainees with adjustment disorders benefitted from this innovative path forward in correctional healthcare. Further research is needed to improve the PDC's service for female pre-trial detainees.

## Introduction

In Europe, 1 in 4 people will meet the diagnostic criteria for a mental illness in their lifetime (1), with an annual prevalence of 15–20% (2, 3). At particularly high risk are individuals in correctional settings, with international research consistently finding increased rates of major groups of mental disorders compared to the general population (4–6). With regards to specific diagnoses, there is some variability between studies, samples and countries. However, systematic reviews and meta-analyses reveal that substance use disorders with prevalence rates between 18 and 30% (7) occur up to six times (8), personality disorders with prevalence rates of up to 65% (9) approximately six times (10), psychotic disorders with 4% prevalence (5) more than ten times (11), major depression with a prevalence of about 11% (5) almost three times (12), and attention-deficit/hyperactivity disorders with slightly more than 25% prevalence at least five times more often in justice-involved persons compared to the general population (13, 14).

Despite the global literature base in this area, research exploring the prevalence of mental illness in Swiss correctional settings is scarce. A systematic search for peer-reviewed primary studies published through March 2022 using PubMed, PsycINFO, and PSYNDEX^1^ identified only 12 such investigations (Table 1). In the three studies which examined total correctional cohorts in Switzerland, overall prevalence rates were found to be higher than those of the general population for adults in pre-trial detention [(26); *N* = 2,195], adults in penitentiaries [(24); *N* = 1,664], and adolescents in pre-trial detention [(16); *N* = 122]. Compared to general population controls, adults in pre-trial detention had noticeably higher rates of substance abuse disorders, personality disorders, and adjustment disorders, whereas adults in penitentiaries had noticeably higher rates of not just substance abuse disorders and personality disorders but also delusional disorders and stress-related disorders. The prevalence of mental illness for adolescents in pre-trial detention was found to be of particular concern, with 90% diagnosed with a behavioral or emotional condition, including substance abuse disorders, stress-related disorders, anxiety disorders, and mood disorders. Such findings underscore the need for adequate access to mental healthcare services in pre-trial as well as in penitentiary settings in the country.

**Table 1:** Peer-reviewed studies on the prevalence of mental health disorders among imprisoned persons in Switzerland.

**Study**	**Population**	**Canton**	***N***	**Assessment method**	**Prevalence of mental health conditions**	
Augsburger et al. (15)	Imprisoned female offenders in penitentiary for ≥4 weeks	Vaud	60	Review of medical records + Clinical interviews + Mental health screening scales	Any mental health problems	43.3%
					Illicit substance use	49.2%
					Severe anxiety symptoms	30.0%
					**Severe depressive symptoms**	**20.0%**
Bessler et al. (16)	Total cohort of adolescents in pre-trial or security detention	Zurich	122	Review of expert opinions	ICD-10: Fx	90.2%
					ICD-10: F1	64.8%
					ICD-10: F3	28.7%
					ICD-10: F41	32.8%
					ICD-10: F43.1	14.0%
					ICD-10: F9	80.3%
Eytan et al. (17)	Persons in pre-trial detention treated by the medical service	Geneva	1,510	Review of medical records + ICPC-2	Any symptoms of a mental health disorder	45.8%
Gisin et al. (18)	Imprisoned adolescents with ≥1 psychiatric consultation in the past year	Geneva	118	Review of expert opinions	ICD-10: Fx	88%
					ICD-10: F91	29.0%
					ICD-10: F12	32.3%
					ICD-10: F10	25.8%
					ICD-10: F60/61	25.8%
					ICD-10: F43.2	19.4%
Haller et al. (19)	Imprisoned adolescents seen at least once by a physician	Geneva	195	Review of medical records + ICPC-2	Any mental health or substance use problem	87.2%
					Alcohol abuse	26.2%
					Cannabis abuse	31.3%
					Adolescent behavior symptoms/complaints	22.6%
					Acute stress reaction	17.4%
					Feeling anxious/nervous/tense	14.4%
					Depressive disorder	8.7%
					Personality disorder	6.7%
					Psychosis or psychotic symptoms	3.6%
Heller et al. (20)	Imprisoned adolescents in intensive interdisciplinary care in a medicalized environment	Geneva	86	K-SADS-PL	Conduct disorders	59.4%
					Illicit substance use disorders	58.0%
					Alcohol use disorder	30.4%
					ADHD	23.2%
					Oppositional defiant disorder	18.8%
					Depression	11.6%
					Anxiety disorder	11.6%
Krammer et al. (21)	Female offenders assessed by forensic psychiatric experts	Bern	239	Review of expert opinions	ICD-10: F1	64.7%
					ICD-10: F2	11.9%
					ICD-10: F3	29.2%
					ICD-10: F43	22.2%
					ICD-10: F6	59.9%
Krammer et al. (22)	Imprisoned male offenders with a history of psychiatric treatment	Bern	39	SCL-90-R	ICD-10: F1	57.7%
					ICD-10: F6	69.2%
Krammer et al. (23)	Imprisoned male offenders	Bern	49	IES-R	ICD-11: 6B4	22.5%
Moschetti et al. (24)	Total cohort of imprisoned persons in closed facilities of the Canton of Vaud	Vaud	1,664	Review of expert opinions	ICD-10: F1	26.2%
					ICD-10: F2	5.3%
					ICD-10: F3	2.2%
					ICD-10: F4	15.9%
					ICD-10: F6	16.2%
Urbaniok et al. (25)	Male prisoners in court-ordered treatment or pre-trial detention	Zurich	86	PDS	PTSD	27%
Wolff et al. (26)	Total cohort of adult persons leaving pre-trial detention	Geneva	2,195	Review of medical records + ICPC-2	Alcohol abuse	34.8%
					Illicit substance abuse	40.2%
					Psychiatric problems (excl. substance abuse)	16.4%
					Depression	7.4%
					Personality disorder	5.5%
					Adjustment disorder	4.5%
					PTSD	1.0%
					Psychosis	1.0%

*ADHD, Attention-Deficit/Hyperactivity Disorder; ICD-10, International Statistical Classification of Diseases and Related Health Problems, 10th Revision (27); ICD-10: Fx, any mental or behavioral disorder referring to ICD-10; ICD-10: F1, Mental and behavioral disorders due to psychoactive substance use; ICD-10: F2, Schizophrenia, schizotypal, and delusional disorders; ICD-10: F3, Mood (affective) disorders; ICD-10: F32, Mood (affective) disorders, depressive episodes; ICD-10: F4, Neurotic, stress-related, and somatoform disorders; ICD-10: F40, Phobic anxiety disorders; ICD-10: F41, Other anxiety disorders; ICD-10: F43, Reaction to severe stress, adjustment disorders; ICD-10: F43.1/ICD-11: 6B4, Posttraumatic stress disorder; ICD-10: F6, Disorders of adult personality and behavior; ICD-10: F9, Behavioral and emotional disorders with onset usually occurring in childhood and adolescence; ICPC-2, International Classification of Primary Care, Second Edition (28); IES-R, Impact of Event Scale, Revised (29); K-SADS-PL, Kiddie Schedule for Affective Disorders and Schizophrenia, Present and Lifetime (30); PDS, Posttraumatic Diagnostic Scale; PTSD, Posttraumatic stress disorder; SCL-90-R, Symptom Checklist, Revised (31)*.

International human rights organizations like the United Nations or the Council of Europe have established that “prisoners should enjoy the same standards of health care that are available in the community” [(32), p. 8] and “All necessary medical, surgical and psychiatric services, including those available in the community, shall be provided to the prisoner for that purpose [(33), art. 40.2 and 40.5]. Though need for treatment is recognized in criminal justice settings and mental health care is generally provided, there are great barriers to establish equivalence. For example, there is no free choice of psychiatric expert or access to the whole range of treatment options provided to individuals in the community including the setting of treatment. While in Swiss criminal justice settings psychiatric care is typically delivered *via* intra-institutional primary psychiatric care (i.e., psychiatric consultation hours on demand either by an external provider or internal psychiatric staff) and inpatient treatment in psychiatric hospitals [for those individuals suffering from an acute mental crisis; (34)], the care system in the community offers further treatment options, e.g., day clinics which have strongly gained in importance in the course of psychiatric reforms in the mid-20th century, that aimed at deinstitutionalizing (35). Day clinics offer multimodal treatment in a setting similar to patients' usual environments and enable to address personal resources as well issues and conflicts typical to the patient's environment (35, 36). They are suitable for patients who do not sufficiently benefit from outpatient treatment but for whom placement in a psychiatric hospital would exceed their actual treatment needs or may be ineffective. There are several models of day clinics which focus on different aspects of treatment. These models can be summarized into the following categories: Day clinics which provide treatment in acute crises either as alternative to inpatient treatment or extension of outpatient treatment (acute treatment), day clinics that support transition from inpatient treatment to the community (rehabilitation) and day clinics that provide long-term support to settle in the society incl. to manage work and social contacts (chronic care) (35, 37). Several randomized controlled trials have shown that day clinics conceptualized to offer an alternative to inpatient treatment are as effective as inpatient treatment (38–40). Research also indicates that patients with moderate symptoms (39) or diagnosed with affective or anxiety disorders (41) particularly benefit from treatment in day clinics. On the basis of a meta-analysis on nine randomized controlled trials (42) estimated that about one quarter of individuals admitted to psychiatric hospitals could actually effectively be treated in acute day clinics. Additionally, research suggests that acute psychiatric day clinics are appropriate from a cost-benefit perspective as it is less expensive (20–45%) for the same level of effectiveness (37, 38, 42).

Little is currently known about day clinics in criminal justice settings. Few experiences are published on on-site day clinics in penitentiaries of the Netherlands and Germany (43, 44), both providing treatment to mitigate acute crises. To our knowledge information is lacking for pre-trial detention completely. Pre-trial detention, however, is argued to be the most stressful period of imprisonment (45–47) when individuals were just been torn out of everyday life, are confronted with uncertainty about further proceedings of their case and still have to adapt to particular restrictive conditions of imprisonment (due to securing the criminal proceedings) compared to those of penitentiaries. The vulnerability of individuals in pre-trial detention (hereinafter “detainees”) is reflected by a higher rate of mental disorders compared to individuals in penitentiaries [hereinafter “prisoners”; (48, 49)] as well as their particularly high suicide rates (50, 51). In Switzerland, they account for more than half of all suicides in prisons although representing only about one third of the Swiss inmate population (52–56). Hence, in 2019, the Canton of Zurich opened an on-site psychiatric day clinic (PDC) to provide intensive treatment services to detainees and fill the gap between intra-institutional primary care consultations and external hospital admissions. It was aimed to care for individuals at risk of acute crisis so as to avoid hospitalization and also, based on the principle of equivalence, provide resources for mentally burdened detainees who could yet not adequately be addressed by the previous treatment options. Detainees are admitted on a voluntary basis and upon recommendation of the attending psychiatrist of primary psychiatric care. The individual's voluntariness is understood to be one important condition for the success of an intervention in the PDC. The new PDC's four full-time nurses and one full-time psychiatrist simultaneously serve nine detainees at a time (57), with the sole admission criteria for the clinic being treatment compliance and exclusion criteria including acute risk of self-harm, acute risk of violence toward others, current psychotic episodes, and deprivation. Besides its comparably high health care resources, the PDC is characterized by more mobility, more access to job activities, sports, and education as well as extensive group activities compared to the usual setting of pre-trial detention (58, 59). Different from day clinics in the community, patients stay in the PDC also during nights, as everyday transfers from pre-trial detention centers to the PDC are not feasible. The PDC was the first of its kind in Switzerland, though similar facilities have since opened in a pre-trial detention center in the Canton of Basel-Stadt in 2021 (60) and a penitentiary in the Canton of Bern in 2022 (61).

Although research from non-correctional settings in Switzerland and other countries has found that PDCs provide effective mental health services, no such research has been conducted exploring the effectiveness of PDC services for pre-trial detainees.

### Study aim and research questions

The aim of the present cross-sectional observational study was to explore three research questions: (1) Is the use of mental healthcare services more frequent among pre-trial detainees than sentenced prisoners? (2) Did the opening of the PDC result in a reduction in psychiatric hospitalizations among pre-trial detainees? (3) Do pre-trial detainees treated exclusively in the PDC differ systematically from pre-trial detainees (also) admitted to psychiatric hospitals?

## Methods

### Setting

The correctional system of Switzerland is decentralized, operating separately in each of its 26 cantons. In the country's most populous canton, the Canton of Zurich, seven pre-trial detention centers and five penitentiaries are currently operated by the Office of Corrections and Rehabilitation of the Canton of Zurich (OCR). Pre-trial detention is carried out with a total capacity of currently 408 places [Justizvollzug und Wiedereingliederung; (62)] corresponding to ~1,800 admissions each year (63). In the Canton of Zurich pre-trial detainees as well as sentenced prisoners have access to psychiatric care in various ways. Primary outpatient psychiatric care is provided by an external agency *via* psychiatric consultations on demand—at the own request of the imprisoned individual or on indication of prison staff. In acute psychiatric crises which cannot be dealt with within the framework of outpatient primary psychiatric care, individuals are transferred to acute wards of general or forensic psychiatric hospitals. Since February 2019, mainly pre-trial detainees can also be admitted to an on-site PDC, which addresses individuals who do neither sufficiently benefit from outpatient primacy psychiatric care, nor is transfer to an acute ward (yet) an appropriate treatment. The PDC is part of the OCR's Department of Pre-trial Detention and is located at the Limmattal pre-trial detention center. All pre-trial detainees from any of the seven centers have access to its services, as do sentenced prisoners when there is spare capacity. Admissions to the PDC are voluntary. Clinically, admission is based on the recommendation of medical staff in pre-trial detention. Legally, admission is based on the responsibility of the OCR to provide medical and mental health care for pre-trial detainees in need. Taking into account an individual's voluntary desire for admission and considering the previously mentioned exclusion criteria, the head of PDC makes the final decision on admission.

### Study design and samples

The study followed an observational cross-sectional design, with data collected retrospectively from existing records from the OCR on annual occupation rate of pre-trial detention centers and penitentiaries as well as psychiatric consultations, admissions to psychiatric hospitals and admissions to the PDC including diagnosis of a mental and behavioral disorder listed as main reason for intervention and diagnosed by medical staff in charge. To investigate Research Question 1, i.e., whether pre-trial detainees accessed mental healthcare services more often than sentenced prisoners, a total count was taken of primary psychiatric care consultations (*k*_Consultations_ = 24,928) and psychiatric hospital admissions (*k*_Admissions_ = 801) for pre-trial detainees and sentenced prisoners in the 5 years prior to the opening of the PDC (January 1, 2014, to December 31, 2018). Individuals kept in the facility responsible for immigration detention were excluded.

To explore Research Question 2, i.e., whether the rate of psychiatric hospital admissions changed for pre-trial detainees before compared to after the opening of the PDC, total counts of all pre-trial detainee hospitalizations were extracted for the respective periods of January 1, 2014, to December 31, 2018 (*k*_Admissions_ = 801) and July 1, 2019, to December 31, 2020 (*k*_Admissions_ = 110).

Finally, to examine Research Question 3, i.e., whether pre-trial detainees treated exclusively in the PDC differ systematically from pre-trial detainees admitted to psychiatric hospitals, data were extracted on all detainees who were admitted in the PDC or a psychiatric hospital between July 1, 2019, and December 31, 2020 (*N* = 115). This sample was divided into three treatment groups: (1) individuals exclusively treated in the PDC (hereinafter “PDC-Only”; *n* = 41, 35.7%), (2) individuals exclusively treated in a psychiatric hospital (hereinafter “Hospital Only”; *n* = 58, 50.4%), and (3) individuals treated in both the PDC as well as a psychiatric hospital (hereinafter “PDC + Hospital”; *n* = 16, 13.9%).^2^

### Statistical analysis

Data were analyzed descriptively, and inferential statistics were calculated to assess group differences, with *t*-tests conducted for continuous variables and Fisher's exact tests or χ^2^ tests conducted for dichotomous variables. To explore changes in psychiatric hospitalization (Research Question 2), total counts of admitted pre-trial detainees in different time periods were calculated in relation to the annual occupancy rate of available spots in the observed pre-trial detention centers and penitentiaries. Such spots cannot be occupied by multiple individuals at the same time; however, in the same year, such spots can be occupied by several individuals, due to releases and new entries. To explore group-level differences between PDC-pre-trial detainees and pre-trial detainees treated elsewhere (Research Question 3) the following information was extracted from file records: *Principal diagnosis* [the ICD-10 (27) diagnosis of a mental and behavioral disorder listed as the main reason for admission to the PDC or a psychiatric hospital], *gender* (0 = male, 1 = female), *age* (continuous, in years) and *alleged index offense* [the reason for current detention in accordance with the articles of the Swiss Criminal Code (64), wherein for analysis, the offenses were combined into the following broader categories: [1] hands-on violent offenses, [2] sexual offenses, [3] threats/extortion/coercion, [4] property crimes, [5] drug-related offenses, and [6] other]. All statistical analyses were performed with Stata 16 SE (65) with a two-tailed significance level of α = 0.05. The proportion of missing values was below 5% for all variables included in the analyses, with missing values excluded from analyses on a case-wise basis.

## Results

### Research question 1: Is the use of mental healthcare services more frequent among pre-trial detainees than sentenced prisoners?

Of the 24,928 primary psychiatric care consultations carried out between 2014 and 2018, 13,478 (54.1%) involved pre-trial detainees and 11,450 (45.9%) involved sentenced prisoners. There was a statistically significant difference between the average annual number of consultations per pre-trial detention spot (*M* = 7.70, *SD* = 1.18) and the average annual number of consultations per penitentiary spot (*M* = 3.43, *SD* = 0.42), *t* = 3.44, *p* < 0.001 (Table 2), i.e., pre-trial detainees are significantly more often accessing primary psychiatric care than sentenced prisoners. More than half (53%) of all individuals in pre-trial detention and penitentiaries treated in primary care were diagnosed with a neurotic, stress-related, or somatoform disorder (ICD-10: F4); 23% had a mental or behavioral disorder due to psychoactive substance use (ICD-10: F1); 6% each had a schizophrenia, schizotypal, or delusional disorder (ICD-10: F2) or a disorder of adult personality or behavior (ICD-10: F6). The principal diagnoses of detainees who received primary psychiatric care differed significantly from those of prisoners, $\chi_{\left(4,321,\;6\right)}^{2}$ = 90.79; *p* < 0.001 (Table 3).

**Table 2:** Annual primary psychiatric care consultations and psychiatric hospital admissions between 2014 and 2018 by type of correctional setting.

**Type of imprisonment**	**Annual number of spots** ***M*** **(*****SD*****)**	**Annual consultations** ***M*** **(*****SD*****)**	**Annual consultations per spots** ***M*** **(*****SD*****)**	**Annual admissions** ***M (SD)***	**Annual admissions per spots** ***M*** **(*****SD*****)**
Pre-trial detention	352.82 (29.33)	2,695.60 (302.99)	7.70 (1.18)	96.0 (26.50)	0.27 (0.08)
Penitentiary	669.30 (12.90)	2,290.00 (262.31)	3.43 (0.42)	64.20 (13.78)	0.10 (0.02)
Total	1,022.11 (37.26)	4,985.60 (522.86)	4.89 (0.62)	160.20 (31.44)	0.16 (0.04)

**Table 3:** Comparison of principal diagnoses in individuals receiving primary psychiatric care between 2014 and 2018 by type of correctional setting (*N* = 4,321).

	**Pre-trial detention**		**Penitentiaries**		**Total**	
**Principal diagnosis**	***n***	**%**	***n***	**%**	***n***	**%**
ICD-10: F1	571	21.0	405	25.4	976	22.6
ICD-10: F2	158	5.8	117	7.3	275	6.4
ICD-10: F3	104	3.8	66	4.1	170	3.9
ICD-10: F4	1,547	56.8	745	46.6	2,292	53.0
ICD-10: F6	102	3.7	147	9.2	249	5.8
ICD-10: F99	187	6.9	79	4.9	2.66	6.2
Other (ICD-10: F0, F5, F7, F8, F9)	55	2.0	38	2.4	93	2.2
Total	2,724	100.0	1,597	100.0	4,321	100.0

*ICD-10: F1, Mental and behavioral disorders due to psychoactive substance use; ICD-10: F2, Schizophrenia, schizotypal, and delusional disorders; ICD-10: F3, Mood [affective] disorders; ICD-10: F4, Neurotic, stress-related and somatoform disorders; ICD-10: F6, Disorders of adult personality and behavior; ICD-10 F99, Unspecified mental disorder*.

Of the 801 psychiatric hospital admissions made between 2014 and 2018, 480 (59.9%) were pre-trial detainees and 321 (40.1%) were sentenced prisoners. There was a statistically significant difference between the average annual number of admissions per pre-trial detention spot (*M* = 0.27, *SD* = 0.08) and the average annual number of admissions per penitentiary spot (*M* = 0.10, *SD* = 0.02), *t* = 25.24, *p* < 0.001 (Table 2), i.e., pre-trial detainees are significantly more often admitted to psychiatric hospitals than sentenced prisoners. Of the individuals admitted to psychiatric hospitals, 40% were diagnosed with schizophrenia, schizotypal, or delusional disorders (ICD-10: F2, *n* = 195, 41.9%), followed by one-third admitted because of neurotic, stress-related, and somatoform disorders (ICD-10: F4, *n* = 151, 32.5%). The principal diagnoses of pre-trial detainees who were admitted to psychiatric hospitals differed significantly from those of sentenced prisoners, $\chi_{\left(465,\;5\right)}^{2}$ = 13.81; *p* < 0.017 (Table 4).

**Table 4:** Comparison of principal diagnoses in individuals admitted to psychiatric hospitals for crisis intervention between 2014 and 2018 by type of correctional setting (*N* = 465).

	**Pre-trial detention**		**Penitentiaries**		**Total**	
**Principal diagnosis**	***n***	**%**	***n***	**%**	***n***	**%**
ICD-10: F1	17	6.2	8	4.1	25	5.4
ICD-10: F2	124	45.3	71	37.2	195	41.9
ICD-10: F3	24	8.8	14	7.3	38	8.2
ICD-10: F4	88	32.1	63	33.0	151	32.5
ICD-10: F6	18	6.6	28	14.7	46	9.9
Other (ICD-10: F0, F5, F7, F8, F9)	3	1.1	7	3.7	10	2.2
Total	274	100.0	204	100.0	465	100.0

*Although there were 480 admissions to psychiatric hospitals for individuals in pre-trial detention between 2014 and 2018, no information about psychiatric diagnosis was available for 15 of them, resulting in a sample size of N = 465. ICD-10: F1, Mental and behavioral disorders due to psychoactive substance use; ICD-10: F2, Schizophrenia, schizotypal, and delusional disorders; ICD-10: F3, Mood (affective) disorders; ICD-10: F4, Neurotic, stress-related, and somatoform disorders; ICD-10: F6, Disorders of adult personality and behavior*.

### Research question 2: Did the opening of the PDC result in a reduction in psychiatric hospitalizations among pre-trial detainees?

Before the PDC started operating in 2019, 1 admission to a psychiatric hospital was registered for almost every 3rd occupied spot in pre-trial detention (0.27 per occupied pre-trial detention spot). After regular operation of the PDC began, this was reduced to 1 admission for almost every 5th occupied pre-trial detention spot (0.22 per occupied pre-trial detention spot). In other words, 27 hospital admissions per 100 pre-trial detention spots decreased to 22 after the PDC started operating. This represents a statistically significant reduction of 18.5% in psychiatric hospital admissions [*t*_(90)_ = 6.46, *p* < 0.001].

### Research question 3: Do detainees treated exclusively in the PDC differ systematically from detainees (also) admitted to psychiatric hospitals?

PDC-Only pre-trial detainees were found to differ statistically from Hospital-Only pre-trial detainees (Table 5). With regards to their principal psychiatric diagnosis, PDC-Only pre-trial detainees were significantly less likely than Hospital-Only pre-trial detainees to be diagnosed with the ICD-10: F2 conditions of schizophrenia, schizotypal disorder, or delusional disorders (*n*_PDC−Only_ = 3, 7.3%; *n*_Hospital−Only_ = 33, 57.9%), $\chi_{\left(98,\;1\right)}^{2}$ = 26.25, *p* < 0.001. PDC-Only pre-trial detainees were however significantly more likely than Hospital-Only detainees found to be diagnosed with the ICD-10: F4 conditions of neurotic, stress-related, or somatoform disorders (*n*_PDC−Only_ = 22, 53.7%; *n*_Hospital−Only_ = 10, 17.5%), $\chi_{\left(98,\;1\right)}^{2}$ = 14.14, *p* < 0.001. With regards to gender, no females were treated exclusively in the PDC (*n*_PDC−Only_ = 0, 0%), whereas ~1 in 8 of pre-trial detainees treated exclusively in psychiatric hospitals were female (*n*_Hospital−Only_ = 8, 13.8%), *p*[Fisher's exact test] = 0.019. Finally, although there were no statistically significant group-level differences in alleged index offenses, descriptive analyses revealed a trend toward more PDC-Only pre-trial detainees having been accused of a sexual offense (*n*_PDC−Only_ = 9, 22.0%) than were Hospital-Only pre-trial detainees (n_*Hospital*−*Only*_ = 4, 6.9%).

**Table 5:** Characteristics of individuals in Zurich's pre-trial detention centers treated in clinical settings between 1st July 2019 and 31st December 2020 (*N* = 115).

	**PDC-only (*****n*** = **41)**		**Hospital-only (*****n*** = **58)**		**PDC** + **hospital (*****n*** = **16)**	
	***n***	**%**	***n***	**%**	***n***	**%**
**Principal diagnosis**						
ICD-10: F1	4	9.8	4	7.0	1	6.3
ICD-10: F2	3	7.3	33	57.9	10	62.5
ICD-10: F3	10	24.4	9	15.8	1	6.3
ICD-10: F4	22	53.7	10	17.5	3	18.8
ICD-10: F6	–	–	–	–	–	–
Other	2	4.9	1	1.8	1	6.3
Gender	41		58		16	
Female	0	0.0	8	13.8	3	18.8
Male	41	100.0	50	86.2	13	81.3
Age in years (*M, SD*)	34.4	8.7	34.5	11.3	36.4	13.5
**Alleged index offense**						
Violent offense (hands-on)	10	24.4	18	31.0	12	75.0
Sexual offense	9	22.0	4	6.9	1	6.3
Threat, extortion, coercion	8	19.5	16	27.6	1	6.3
Property crime	7	17.1	12	20.7	1	6.3
Drug-related offense	2	4.9	2	3.4	–	–
Other	5	12.2	6	10.3	1	6.3

*PDC, Psychiatric day clinic; ICD-10: F1, Mental and behavioral disorders due to psychoactive substance use; ICD-10: F2, Schizophrenia, schizotypal, and delusional disorders; ICD-10: F3, Mood (affective) disorders; ICD-10: F4, Neurotic, stress-related, and somatoform disorders; ICD-10: F6, Disorders of adult personality and behavior*.

**Figure 1:**
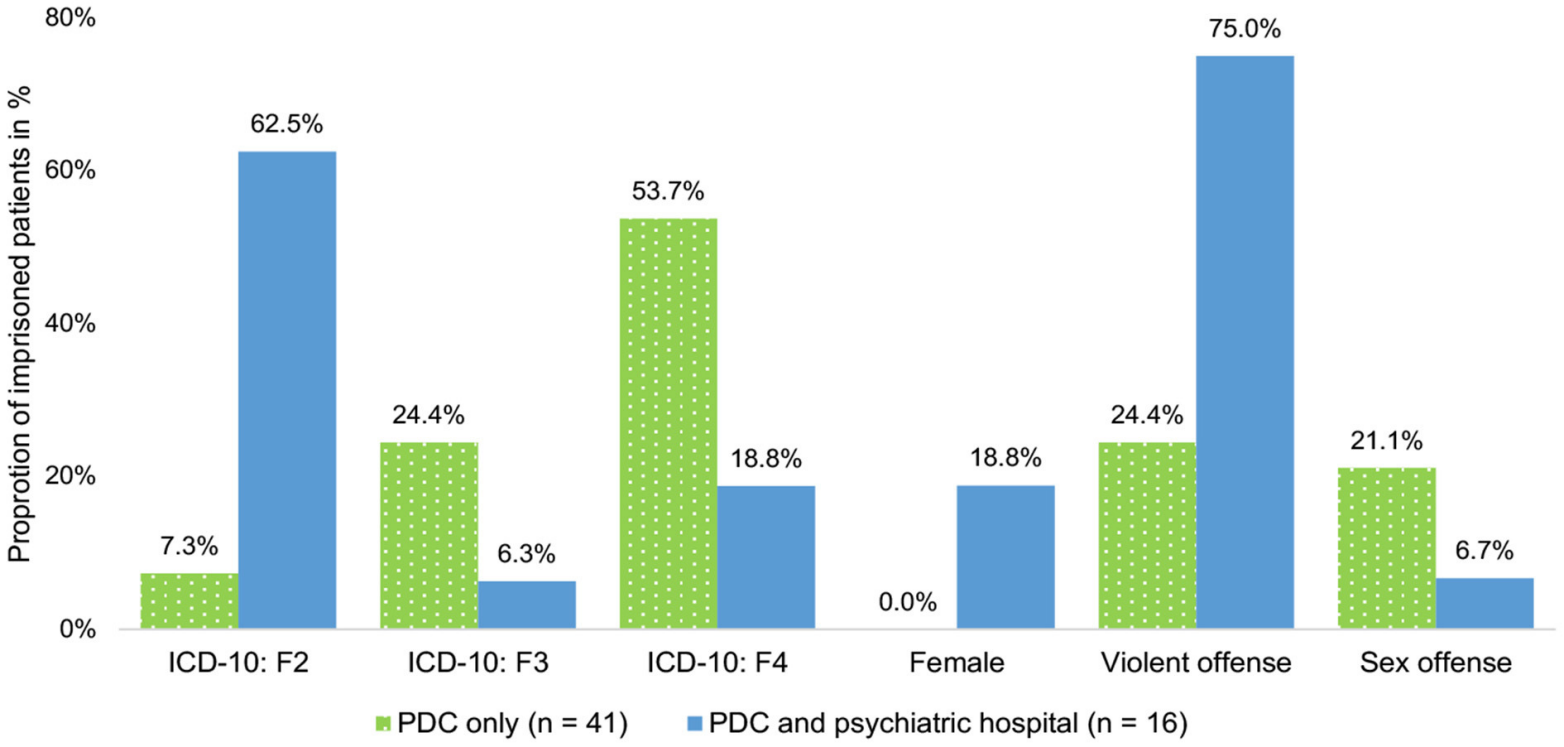
Comparing characteristics of individuals treated only in the psychiatric day clinic with patients treated in both the psychiatric day clinic as well as a psychiatric hospital.

Comparisons between PDC-Only pre-trial detainees and PDC + Hospital pre-trial detainees did also reveale significant differences in principal psychiatric diagnoses (*p*[Fisher's exact test] <0.001), gender (*p*[Fisher's exact test] = 0.019), and alleged index offenses (*p*[Fisher's exact test] = 0.036) see (Figure 1). With regards to their principal psychiatric diagnosis, ~2 in 3 of the PDC + Hospital pre-trial detainees were diagnosed with the ICD-10: F2 conditions of schizophrenia, schizotypal disorder, or delusional disorders (*n*_PDC+Hospital_ = 10, 62.5%) compared to <1 in 10 of the PDC-Only pre-trial detainees (*n*_PDC−Only_ = 3, 7.3%). However, PDC-Only pre-trial detainees were approximately three-times as likely to be diagnosed with ICD-10: F4 conditions of neurotic, stress-related, or somatoform disorders compared to PDC + Hospital pre-trial detainees (*n*_PDC−Only_ = 22, 53.7%; *n*_PDC+Hospital_ = 3, 18.8%). Furthermore, PDC-Only pre-trial detainees were approximately four-times as likely to be diagnosed with the ICD-10: F3 affective disorders compared to PDC + Hospital pre-trial detainees (*n*_PDC−Only_ = 10, 24.4%; *n*_PDC+Hospital_ = 1, 6.3%). In terms of gender, ~1 in 5 pre-trial detainees in the PDC + Hospital group were female (*n*_PDC+Hospital_ = 3, 18.8%) compared to none of the detainees in the PDC-Only group. Finally, the majority of PDC + Hospital pre-trial detainees were accused of hands-on violent offenses (*n*_PDC+Hospital_ = 12, 75.0%) compared with only a quarter of the PDC-Only pre-trial detainees (*n*_PDC−Only_ = 10, 24.4%).

## Discussion

Psychiatric day clinics represent an evidence-based middle ground between low intensity psychiatric primary care and high intensity psychiatric hospitalization for individuals diagnosed with mental illnesses. Despite a growing literature on their effectiveness for general population controls (37, 38, 42), PDCs for justice-involved persons, who research has shown are particularly at risk for mental illness (5, 6), are rare, and to our knowledge no studies exist on such clinics' effectiveness for pre-trial detainees—a particularly vulnerable prison population. The aim of the present study was to address this gap by reviewing Canton of Zurich medical records for pre-trial detainees and sentenced prisoners both before and after a PDC began its operations in 2019.

First, the prevalence of primary psychiatric care consultations and psychiatric hospital admissions was assessed in the 5 years before the opening of the PDC, with pre-trial detainees being found to receive both more consultations and hospitalizations for crisis intervention relative to sentenced prisoners. For both correctional groups, the most common diagnoses cited as a cause for psychiatric consultation were neurotic, stress-related, or somatoform disorders (ICD-10: F4), followed by substance use disorders (ICD-10: F1). Regarding hospitalizations, both pre-trial detainees and sentenced prisoners were primarily admitted because of schizophrenia spectrum disorders (ICD-10: F2), followed by neurotic, stress-related, or somatoform disorders. The prevalence of adult disorders of personality and behavior was lower in pre-trial detainees [4% (primary care) and 7% (hospitalization)] than in sentenced prisoners [9% (primary care) and 15% (hospitalization)] and generally lower than rates reported in previous prison research [e.g., (66, 67)]. However, this may be due to the fact that information needed to diagnose such disorders was largely unavailable due to reliance on self-reports and rather short observation times. Moreover, adult disorders of personality and behavior may lead to acute psychiatric treatment less often than other disorders and thus, may be less often detected.

Second, the impact of an on-site acute psychiatric day clinic in pre-trial detention in the Canton of Zurich was examined. The goal of the PDC is to facilitate access to multimodal mental health care in the environment of pre-trial detention but under less restrictive conditions to prevent severe emotional crises which would otherwise require psychiatric hospitalization. Findings of the present study suggest that the PDC has positively impacted the provision of mental healthcare services during pre-trial detention, with hospital admissions decreasing by 18.5% in the clinic's first 18 months of operation. Furthermore, three of four (71.9%) pre-trial detainees admitted to the PDC did not require more intensive treatment.

Third, PDC admissions were primarily for individuals diagnosed with neurotic, stress-related, or somatoform disorders followed by affective disorders. Individuals with such diagnoses are believed to be those most helped by PDCs, given that day clinic services are offered in an environment similar to the pre-trial detainee's current living situation with a focus on developing coping skills (38). The findings of the present study suggest that individuals admitted to the PDC differ systematically from those who are admitted to a psychiatric hospital (i.e., mainly detainees with schizophrenia spectrum disorders), suggesting a unique subgroup of pre-trial detainees who benefit from treatment but are not typically transferred for inpatient hospital treatment. In this context, it may be of specific interest to service planners that there appears to be only a very small group of pre-trial detainees with chronic psychotic illnesses who are too unwell to gain maximum benefit from primary psychiatric care but not so unwell to warrant transfer to an external hospital. This may indicate that mild crises which are manageable in an institution such as the PDC are rare amongst individuals with psychotic illnesses.

None of the female pre-trial detainees, however, were exclusively treated in the PDC but were (additionally) admitted to psychiatric hospitals, suggesting that the treatment services available in the day clinic were judged as not sufficient to meet their healthcare needs. One reason for this may be a higher burden of severe mental disorders amongst female offenders compared to male offenders (68, 69), making them in need of the acute crisis care provided in psychiatric hospitals. That said, it is also possible that mental healthcare simply represents one of many areas of the criminal justice system in need of gender-responsive adaptations to produce the most positive outcomes for women offenders (70).

In summary, the establishment of the PDC provides an innovative path to improve mental health care in pre-trial detention. Not only has the spectrum of provided interventions been expanded, but it also addressed a specific group of pre-trial detainees for which adequate treatment previously seemed unavailable. In addition, the new service has a positive effect on the prison system, due to the PDC being embedded within the existing correctional system: Transfer to the PDC and back to custody is easier, more immediate, and requires fewer resources than external services, and it also improves the continuity and immediacy of mental health care.

## Limitations

There are four principal limitations of the present investigation. First, psychiatric hospital admission rates for pre-trial detainees as well as group differences between pre-trial detainees treated only in the PDC vs. in a psychiatric hospital were evaluated only 18 months after the PDC began operating. This relatively short observation period, in which a small sample of only 57 pre-trial detainees were admitted to the new clinic, necessitate viewing the findings of the present study as preliminary and in need of future replication.

Second, it was not possible to explore the reasons why pre-trial detainees of the PDC were also admitted to a psychiatric hospital, as the file records which were reviewed as part of this study lacked information about the course of treatment. Thus, we were not able to identify the reason for additional treatment, i.e., if pre-trial detainees were truly misplaced in the PDC or if placement in the PDC was chosen intermediately due to the lack of available beds in a psychiatric hospital. Hence, it is possible that different reasons for admission could be a moderating factor for the identified rate or group differences, making it important that future research collect and subsequently incorporate such information into analyses.

Third, prevalence rates of mental disorders were calculated on the basis of primary psychiatric care consultations, psychiatric hospital admissions, as well as PDC admissions. Thus, the collected data demonstrates the prevalence of mental disorders among individuals in correctional settings who are in need of acute treatment. Hence, the data may not accurately convey the true prevalence of mental disorders in the prison population of the Canton of Zurich. Due to the lack of standardized diagnostic assessment at time of prison admission, this information is yet not available in the Canton Zurich.

Fourth, minority ethnic groups are overrepresented in prisons world-wide and there is some evidence that this group has restricted access to healthcare in custody. Unfortunately, we had no access to valid information on ethnicity or racial identity, as nationality was the only variable recorded in the analyzed data. However, we do not consider nationality to be a valid representation of ethnicity or racial identity as, e.g., former immigrants with current Swiss nationality may still identify with a different ethnicity or racial identity or are still perceived to have a different racial identity.

## Conclusion and practical implications

Empirically exploring innovations in mental healthcare is critical to the welfare of our communities, and this is particularly true for justice-involved persons, who are particularly at risk for developing a mental disorder. The current article presented the findings of the first research investigation of a total cohort of justice-involved persons in Switzerland undergoing different forms of psychiatric care. The findings suggest that the need for evidence-based mental healthcare services is higher in pre-trial detention settings relative to prison settings. To meet this need, an on-site PDC serving pre-trial detainees was found to complement primary psychiatric care services and psychiatric hospital admissions. The PDC appears to be especially beneficial for pre-trial detainees diagnosed with adjustment, stress, anxiety, and affective disorders for who primary psychiatric care alone is not always sufficient but for who hospital admission may be excessive. Our preliminary data does not support the admission of pre-trial detainees diagnosed with schizophrenia spectrum disorders to the PDC, and the clinic's services need to be improved for women pre-trial detainees, as they have not benefitted from the new site yet.

## Data availability statement

The anonymized raw data supporting the conclusions of this article will be made be available by request to the corresponding author.

## Ethics statement

The Ethics Committee of the Canton of Zurich waived ethics approval for the present study (Req-2021-00290), as it is not subject to Human Research Act. The use of file data for the current study was approved by the data protection officer of the OCR. Written informed consent for participation was not required for this study in accordance with the national legislation and the institutional requirements.

## Author contributions

JG, JE, MW, and AR designed the study. JG and MW prepared the data. Supervised by JG and AR, MW performed the statistical analyses. JG took the lead in writing the manuscript in close collaboration with JE, AR, MW, MG, and JS. All authors provided critical feedback and helped shape analyses and interpretation. All authors contributed to the article and approved the submitted version.

## Conflict of interest

JS was employed by Publication Academy Inc.

The remaining authors declare that the research was conducted in the absence of any commercial or financial relationships that could be construed as a potential conflict of interest.

## Publisher's note

All claims expressed in this article are solely those of the authors and do not necessarily represent those of their affiliated organizations, or those of the publisher, the editors and the reviewers. Any product that may be evaluated in this article, or claim that may be made by its manufacturer, is not guaranteed or endorsed by the publisher.

## Footnotes

^1^The following search terms [title or abstract] were applied: (mental health OR mental illness OR mental disorder OR psychiatric illness) AND (prison^*^ OR detention OR inmates OR incarcerated) AND (Switzerland OR swiss). This search strategy resulted in 64 hits (PubMed: *n* = 32; PsycINFO: *n* = 25; PSYNDEX: *n* = 7). After removing 18 duplicate records, 34 of the remaining 46 unique hits were excluded as they either reported on the same sample as a previous study, did not report prevalence rates of mental disorders in a prison sample, or did not report on a prison sample in Switzerland.

^2^If individuals were hospitalized more than once within the observed period, only the first hospitalization was included to avoid correlated data.
